# Endometrial thickness affects the outcome of in vitro fertilization and embryo transfer in normal responders after GnRH antagonist administration

**DOI:** 10.1186/1477-7827-12-96

**Published:** 2014-10-09

**Authors:** Yu Wu, Xiaohong Gao, Xiang Lu, Ji Xi, Shan Jiang, Yin Sun, Xiaowei Xi

**Affiliations:** Reproductive Medicine, Department of Obstetrics and Gynecology, Shanghai Jiaotong University Affiliated First People’s Hospital, No. 650, New Songjiang Road, Shanghai, 201620 China; Department of Reproductive Medicine, Shanghai Jiaotong University International Peace MCH Hospital, No. 910, Henshan Road, Shanghai, 200030 China

**Keywords:** Endometrial thickness, IVF-ET, GnRH antagonist, Pregnancy rate

## Abstract

**Background:**

The goal of this study was to assess the association between endometrial thickness on the chorionic gonadotropin (hCG) day and in vitro fertilization and embryo transfer (IVF-ET) outcome in normal responders after GnRH antagonist administration.

**Methods:**

A retrospective cohort study was performed in normal responders with GnRH antagonist administration from January 2011–December 2013. Patients were divided into four groups according to endometrial thickness, as follows: <7 mm (group 1), > = 7- < 8 mm (group 2), > = 8- < 14 mm (group 3), and > =14 mm (group 4).

**Results:**

A total of 2106 embryo transfer cycles were analyzed. The pregnancy rate (PR) was 44.87%.

The clinical pregnancy rate, ongoing pregnancy rate and the implantation rate (17.28%, 13.79%, 10.17%, respectively) were significantly lower in group 1 compared to the other three groups (p < 0.05). The miscarriage rate was higher in patients with endometrial thickness less than 7 mm. The clinical pregnancy rate, ongoing pregnancy rate and implantation rate were highest in patients with endometrial thickness higher than 14 mm, but showed no difference in patients with those of endometrial thickness between 8-14 mm.

**Conclusions:**

There is a correlation between endometrial thickness measured on hCG day and clinical outcome in normal responders with GnRH antagonist administration. The pregnancy rate was lower in patients with endometrial thickness less than 7 mm compared with patients with endometrial thickness more than 7 mm.

## Background

In-vitro fertilization (IVF) and intracytoplasmic sperm injection (ICSI) are widely accepted as effective treatment for most causes of infertility. Gonadotropin-releasing hormone (GnRH) antagonist is now widely used in controlled ovarian stimulation cycles, which requires less follicle stimulation, and has lower risk for ovarian hyperstimulation syndrome (OHSS) [1, 2]. A randomized controlled trial (RCT) showed that normal responders treated with the GnRH antagonist protocol exhibited the same high success rates as patients treated with the long GnRH agonist protocol. The GnRH antagonist protocol is as effective and safe as the long GnRH agonist protocol [3].

Age, quality of the embryo and endometrial receptivity are the most important factors for the success of IVF. Endometrial thickness (EMT) has been accepted as an indicator for endometrial receptivity, and assessment of the endometrium in the midsagittal plane via transvaginal ultrasound is the standard procedure. Several studies have shown a significant correlation between pregnancy rate and endometrial thickness [4–7]. These studies reported a threshold of <7 mm with a significant reduction in the implantation rate and pregnancy rate. Recently the first systematic review and meta-analysis investigated both the independent predictive capacity and the prognosic value of endometrial thickness on pregnancy outcomesafter IVF. This study found that the probability of clinical pregnancy for an endometrial thickness ≤7 mm was significantly lower compared with cases with endometrial thickness >7 mm (23.3% versus 48.1%) and OR was 0.42 (95% CI 0.27–0.67) [5]. There are few studies examining the association between endometrial thickness and clinical outcome in the GnRH antagonist protocol. The aim of this study was to assess the association between endometrial thickness on the HCG day and IVF outcome in normal responders after GnRH antagonist administration.

## Methods

This study was reviewed and approved by the Institutional Review Board and Ethics Committee of Shanghai First People’s Hospital, China. This study is a retrospective cohort study and analyzed 2106 normal responders in International Peace MCH Hospital Shanghai Jiaotong University. All fresh embryo transferred patients after GnRH antagonist administration between Jan. 2011 and Dec. 2013 was included except poor responders according to the Bologna criteria [8] and patients with polycystic ovary syndrome (PCOS). Patients with abnormal uterine cavity per HSG or hysteroscopy were excluded from this study.

rFSH/HMG (rFSH-Gonal F-Merck Serono, Puregon-MSD, HMG Lizhu China) was initiated at a dosage of 150 U-225 U per day on day 2 of the cycle. The ovarian response was monitored by ultrasound and serum LH, E2 and P on day 6 (stimulation day 5). The need for additional doses of rFSH/HMG was determined based on follicular maturation, as assessed by ultrasound and E2 measurement. The GnRH antagonist (Cetrotide 0.25 mg-Merck Serono) 0.25 mg/day was added when at least 1 follicle reached 14 mm in diameter until hCG administration. When at least three follicles had reached a diameter of 18 mm, a dose of hCG (hCG, Lizhu China) 6000 U was given and oocyte retrieval was performed 36 hours later using vaginal ultrasound guided follicle aspiration. The endometrial thickness was measured in the midsagittal plane via transvaginal ultrasound on the day of hCG administration. A maximum of 3 embryos were transferred after 2 or 3 days in culture.

Luteal support was administered by intramuscular progesterone (dose 40 mg/day) beginning on the day of oocyte retrieval.

Clinical pregnancy was defined as the presence of at least a gestational sac on ultrasound 28 days after embryo transfer. Ongoing pregnancy was defined as the presence of at least one fetus with heart activity more than 12 gestational weeks. Implantation rate was calculated as the number of sacs with fetal heart beat over total embryos transferred.

All patients were divided into one of the following four groups according to their endometrial thickness: group 1 (n = 29): <7 mm; group 2 (n = 162): ≥7 mm to <8 mm; group 3 (n = 1852): ≥8 mm to <14 mm; group 4 (n = 64): ≥14 mm.

### Statistical methods

Statistical analysis was performed using the statistical package for Social Sciences (version 14, SPSS Inc, Chicago IL). The X2 test, t-test and ANOVA were used. P <0.05 was considered as statistically significant.

## Results

**Figure 1:**
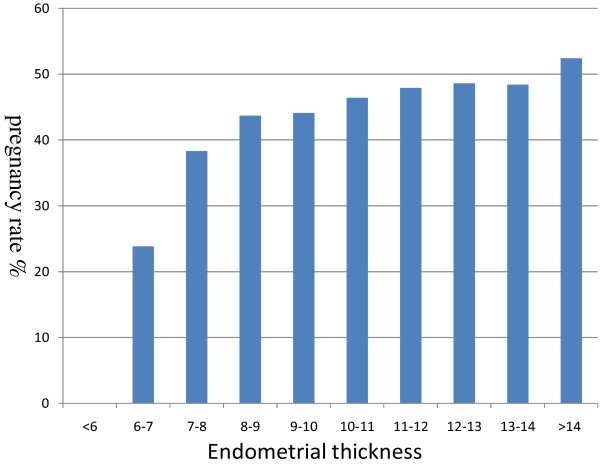
**Clinical pregnancy rates according to endometrial thickness.**

A total of 2106 women aged 21–39 with fresh embryo transfer were included in the analysis. The endometrial thickness on the hCG day ranged from 5 mm to 18.7 mm. The overall pregnancy rate was 44.87%. The clinical pregnancy rate was significantly lower in cases with endometrial thickness below 7 mm. In addition, no pregnancy was observed in the patients with endometrial thickness less than 6 mm (Figure 1). According to the pregnancy rate in Figure 1 (Group1: pregnancy rate <30%, Group2: pregnancy rate 30-40%, Group 3: pregnancy rate 40-50% and Group 4: pregnancy rate >50%), the patients were divided into 4 groups as previously described.

The demographic characteristics of the groups are shown in Table 1. Age, baseline FSH, and baseline E2 were similar among the four groups. More patients in Group 1 had a previous IVF attempt. Identical results were obtained among these groups with regard to the duration of ovarian stimulation, duration of GnRH antagonist administration, and serum E2 concentration on hCG day (Table 2). There were no significant differences among these groups. Moreover, the retrieved oocyte number, transferred embryo number, fertilization rate and cleavage rate were similar among these groups (Table 3).

**Table 1:** **Demographic and pretreatment characteristics**

	Group 1	Group 2	Group 3	Group 4
	N = 29	N = 162	N = 1852	N = 63
**Mean age**	33.12 ± 3.46	32.34 ± 4.02	31.89 ± 3.67	31.98 ± 4.16
**No. of Previous IVF attempts**				
**None**	15(51.72%)*	124(76.54%)	1390(75.05%)	51(80.95%)
**One**	10(34.48%)	30(18.51%)	374(20.19%)	11(17.46%)
**Two or more**	4(13.79%)	8(4.94%)	87(4.70%)	1(1.75%)
**Baseline FSH(U)**	7.84 ± 2.44	8.13 ± 2.12	8.87 ± 5.28	8.89
**Baseline E2 (pmol/L)**	167.71 ± 90.66	184.48 ± 160.48	169.85 ± 253.51	132.88 ± 57.19

*P < 0.05.

**Table 2:** **Outcome of ovarian stimulation**

	Group 1	Group 2	Group 3	Group 4
	N = 29	N = 162	N = 1852	N = 63
**Total dose of Gn**	2137.3	2109.1	2145.6	2089.45
**Duration of Gn**	8.55 ± 1.45	8.52 ± 1.59	8.67 ± 1.49	8.86 ± 1.45
**Duration of antagonist**	3.68 ± 0.81	3.59 ± 1.21	3.67 ± 1.22	3.62 ± 1.24
**Serum E2 on hCG day**	9238 ± 1902.29	10457 ± 5391.63	10166.89 ± 6192.71	9962 ± 4662.25

**Table 3:** **Outcome of pre-embryonic development**

	Group 1	Group 2	Group 3	Group 4
	N = 29	N = 162	N = 1852	N = 63
**IVF/ICSI**	20/9	129/33	1387/475	53/10
**No. of oocyte retrieved**	9.34 ± 6.0	10.78 ± 5.41	10.82 ± 5.60	11.02 ± 4.59
**No. of oocyte fertilized**	7.3	8.0	8.1	8.5
**No. of embryo transferred**	2.03 ± 0.57	2.01 ± 0.39	2.02 ± 0.37	2.07 ± 0.32
**No. of embryo cryopreserved**	2.72 ± 3.68	3.01 ± 2.96	2.97 ± 2.94	2.88 ± 2.89

The clinical pregnancy rate, ongoing pregnancy rate and implantation rate increased with an increase in endometrial thickness. The clinical pregnancy rate, ongoing rate and implantation rate (17.28%, 13.79%, 10.17%, respectively) were lowest in group 1 and were significantly lower than the other three groups (p < 0.05). The miscarriage rate was higher in patients with endometrial thickness less than 7 mm. Among patients with endometrial thickness between 7 mm to 8 mm, the clinical pregnancy rate and implantation rate was lower than that of patients with endometrial thickness more than 8 mm, but this difference was not significant. Only the ongoing pregnancy rate was significantly lower than that in patients with thick endometria (≥14 mm). The clinical pregnancy rate, ongoing rate and implantation rate (52.38%, 47.62%, and 38.17%, respectively) were highest in patients with an endometrial thickness greater than 14 mm (Table 4).

**Table 4:** **Clinical outcome**

	Group 1	Group 2	Group 3	Group 4
	N = 29	N = 162	N = 1852	N = 63
**Clinical pregnant rate**	17.24%*	38.27	45.63%	52.38%
	(5/29)	(62/162)	(845/1852)	(33/63)
**Miscarriage rate**	20%	11.29%	10.30%	9.09%
	(1/5)	(7/62)	(87/845)	(3/33)
**Ongoing rate**	13.79%*	32.10%*	38.23%	47.62%
	4/29	52/162	708/1852	30/63
**Implantation rate**	10.17%*	23.92%	29.80	38.17%

*P < 0.05.

Among 29 patients with thin endometrium (Group 1), 19 patients was secondary infertility before they were included in the study. However the number of the patients with previous IVF attempt in this Group was higher which may have affected the result; nevertheless the pregnancy rate in patients in first cycle was quite low (6.7%, 1/15). The pregnancy rate was 40% among patients in second cycle (4/10), and three of four patients among them who had an endometrial thickness more than 8 mm in previous long GnRH agonist protocol cycle got pregnant in this GnRH antagonist cycle.

## Discussion

Endometrial receptivity is one of the most important factors in predicting pregnancy after in-vitro fertilization and embryo transfer. Endometrial thickness has been utilized as an individual indicator for endometrial receptivity and is measured in the midsagittal plane via transvaginal ultrasound, which is considered as a non-traumatic and a simple method [9]. The endometrial thickness measured on the day of hCG administration is most often used.

The effect of endometrial thickness on the success of IVF-ET had been evaluated by many studies [4–7, 10, 11]. Recent studies have reported a threshold of <7 mm with a significant reduction in the implantation rate and pregnancy rate [5–7]. Meta-analysis showed that the probability of clinical pregnancy in patients with endometrial thickness less than 7 mm was significantly lower compared to patients with endometrial thickness greater than 7 mm [23.3% versus 48.1%, OR 0.42 (95% CI 0.27–0.67)]. Positive and negative predictive values for the outcome of clinical pregnancy were 77% and 48%, respectively [5]. Kumbak also found that the clinical pregnancy rate was 26%, miscarriage rate was 31% and live birth rate was 17% among patients with endometrial thickness less than 7 mm. However, these results were relatively good when the patient age was <35 years or the number of oocytes retrieved was over five or the number of available embryos to transfer was ≥3 [7]. Thus, no conclusive cut-off value of endometrial thickness has been established.

The findings of endometrium thicker than 14 mm are controversial. Several authors suggested a detrimental effect of endometrial thickness of ≥14 mm on pregnancy rate [12], while the results of other studies have suggested that a thick endometrium increased the pregnancy rate [4]. Quintero reported a woman who successfully conceived with an endometrial thickness of 20 mm [13].

Most studies examining the association between endometrial thickness and clinical outcome were performed using the GnRH agonist protocol. With the antagonist protocol being increasingly used, Al-Inany updated the Cochran review in 2011. Forty-five randomized trials (RCT) (n =7511) were included. The results showed that there was no evidence of a statistically significant difference in the rates of live births (OR 0.86, 95% CI 0.69 to 1.08), and there was a statistically significant lower incidence of OHSS in the GnRH antagonist group (OR 0.43, 95% CI 0.33 to 0.57) [14].

The GnRH antagonist was also recently introduced in China. A RCT comparing the antagonist protocol with the agonist long protocol was performed in normal responders. Antagonist treatment with a 43.7% clinical pregnancy rate was more efficient and safer than treatment with a long protocol and demonstrated equally high success rates [3]. In our study, the clinical pregnancy rate was 44.87% in the normal responder with GnRH antagonist administration, which was consistent with the results described above.

There are few studies investigating the association between endometrial thickness and clinical outcome with GnRH antagonist administration. However, these results are controversial [15, 16]. Our study is a retrospective cohort study examining the relationship between endometrial thickness and clinical outcome using the GnRH antagonist protocol in normal responders. These patients were aged 20–39 and patients with PCOS and poor responder were excluded. Our study showed a positive correlation between endometrial thickness and clinical outcome, which is consistent with several studies [6, 16]. There were only five clinical pregnancies (17.24%) in our study, which had an endometrial line of less than 7 mm, of which one was lost. The number of previous IVF attempts was higher in these patients which might affect the pregnancy. Although the pregnancy rate was 6.7% in the patients in first cycle, the high previous IVF attempt is still a limitation of the study. The pregnancy rate among these patients is similar to the pregnancy rate (23.3%) in recently meta-analysis although most women in the meta-analysis were conducted in long GnRH agonist protocol [5].

Patients with an endometrial thickness between 7–8 mm had a decreased pregnancy rate, but no significant difference was shown when compared to patients with endometrial thickness in 8-14 mm.

Implantation is necessary for a successful pregnancy and requires healthy endometrial receptivity [17]. The implantation rate (10.17%) was significantly lower in patients with thin endometrial thickness. Thicker endometria corresponded with higher implantation rates. These findings were consistent with a clinical pregnancy rate and ongoing pregnancy rate results.

In patients with thicker endometria (≥14 mm), the clinical pregnancy rate, ongoing pregnancy rate and implantation rate increased, but with no difference with patients with endometrial thickness in 8-14 mm in our study. This finding was consistent with several recent studies demonstrating no reduction in pregnancy rate with a very thick endometrium [6, 12].

Our study has some limitations, the most important of which is its retrospective nature. However, we believe that the results are of interest because similar studies had been published with conflicting results.

## Conclusions

The results of the present study identified a correlation between endometrial thickness measured on hCG day and clinical outcome in normal responders with GnRH antagonist administration.
